# What Kind of Capsule Endoscope Is Suitable for a Controllable Self-Propelling Capsule Endoscope? Experimental Study Using a Porcine Stomach Model for Clinical Application (with Videos)

**DOI:** 10.1371/journal.pone.0139878

**Published:** 2015-10-08

**Authors:** Kazuhiro Ota, Sadaharu Nouda, Toshihisa Takeuchi, Munetaka Iguchi, Yuichi Kojima, Takanori Kuramoto, Takuya Inoue, Yasunori Shindo, Kenshiro Uesugi, Yoshiaki Fujito, Hironori Nishihara, Naotake Ohtsuka, Kazuhide Higuchi, Team Mermaid

**Affiliations:** 1 2nd Department of Internal Medicine, Osaka Medical College, Takatsuki, Osaka, Japan; 2 Faculty of Science and Technology, Ryukoku University, Seta, Shiga, Japan; 3 Mu Ltd., Seta, Shiga, Japan; Aristotle University of Thessaloniki, GREECE

## Abstract

**Background:**

We have been developing the Self-Propelling Capsule Endoscope (SPCE) that allows for controllability from outside of the body and real-time observation. What kind of capsule endoscope (CE) is suitable for a controllable SPCE is unclear and a very critical point for clinical application. We compared observing ability of three kinds of SPCEs with different viewing angles and frame rates.

**Methods:**

Eleven buttons were sewed in an excised porcine stomach. Four examiners controlled the SPCE using PillCamSB2, -ESO2, and -COLON2 (Given Imaging Ltd., Israel), for 10 minutes each with the aim of detecting as many buttons and examining them as closely as possible. The ability to find lesions was assessed based on the number of detected buttons. The SPCE-performance score (SPS) was used to evaluate the ability to examine the lesions in detail.

**Results:**

The SPCE-ESO2, -COLON2, and -SB2 detected 11 [interquartile range (IQR): 0], 10.5 (IQR, 0.5), and 8 (IQR, 1.0) buttons, respectively. The SPCE-ESO2 and -COLON2 had a significantly better ability to detect lesions than the -SB2 (p < 0.05). The SPCE-ESO2, -COLON2, and -SB2 had significantly different SPS values of 22 (IQR, 0), 16.5 (IQR, 1.5), and 14 (IQR, 1.0), respectively (p < 0.05 for all comparisons; SPCE-SB2 vs. -ESO2, -SB2 vs. -COLON2, and -ESO2 vs. -COLON2).

**Conclusions:**

PillCamESO2 is most suitable in different three CEs for SPCE for examining lesions in detail of the stomach.

## Introduction

Capsule endoscopy is a minimally invasive examination procedure that enables observation of the intestinal tract. A capsule endoscope (CE) moves in the digestive tract by gut peristalsis, and the images it obtains are recorded via wireless communication [1]. Although capsule endoscopy has been employed for the evaluation of the small intestine, esophagus, and colon, which are simple elongated cylindrical organs, it has not been used in the stomach [1–6]. As the stomach comprises a large area with a complex bag shape, it is not possible to observe the whole stomach by gut peristalsis only. Furthermore, in capsule endoscopy, the CE cannot be maintained at a desired position, and unlike with conventional tube-type endoscopy, an examiner cannot observe a lesion from any desired direction [7]. One solution is to append the functions to control from outside of the body and allow for real-time observation to a CE.

We developed the Self-Propelling Capsule Endoscope (SPCE), a CE with the above-mentioned functions. Previously, we achieved control of the SPCE in the stomach of a living dog [8] and safely maneuvered the device in a living human’s stomach, small intestine, and colon [9].

For the practical application of the SPCE, its detecting capability must be assessed objectively. CE functions to allow for controllability from outside of the body and real-time observation have been developed at several institutions [6, 8, and 9]. However, there is no objective report on the detecting capability of such devices. Although we successfully produced a SPCE by attaching a special fin to currently available CEs for the esophagus, small intestine, and colon, each with different viewing angles and frame rates, the most suitable CE for SPCE remained unclear.

For this purpose, we attempted to evaluate the differences in detecting capability and performance among three SPCE types, the fin of which was attached to a PillCamSB2, PillCamESO2, or PillCamCOLON2, in a screening test of the stomach. By comparing the functions of each SPCE type, we sought to clarify the advantage of each function and its feasibility in clinical practice in order to address the critical development problems for any CEs that are controllable from outside of the body in real-time observation, including SPCE.

## Material and Methods

### Driving System for SPCE via the Use of a Magnetic Field (New MiniMermaid System)

We previously reported on the driving system for SPCE via the use of a magnetic field, namely the Ryukoku-Osaka system [8]. In the present study, we used a further modified system, known as the New MiniMermaid system (S1 Video).

**Fig 1:**
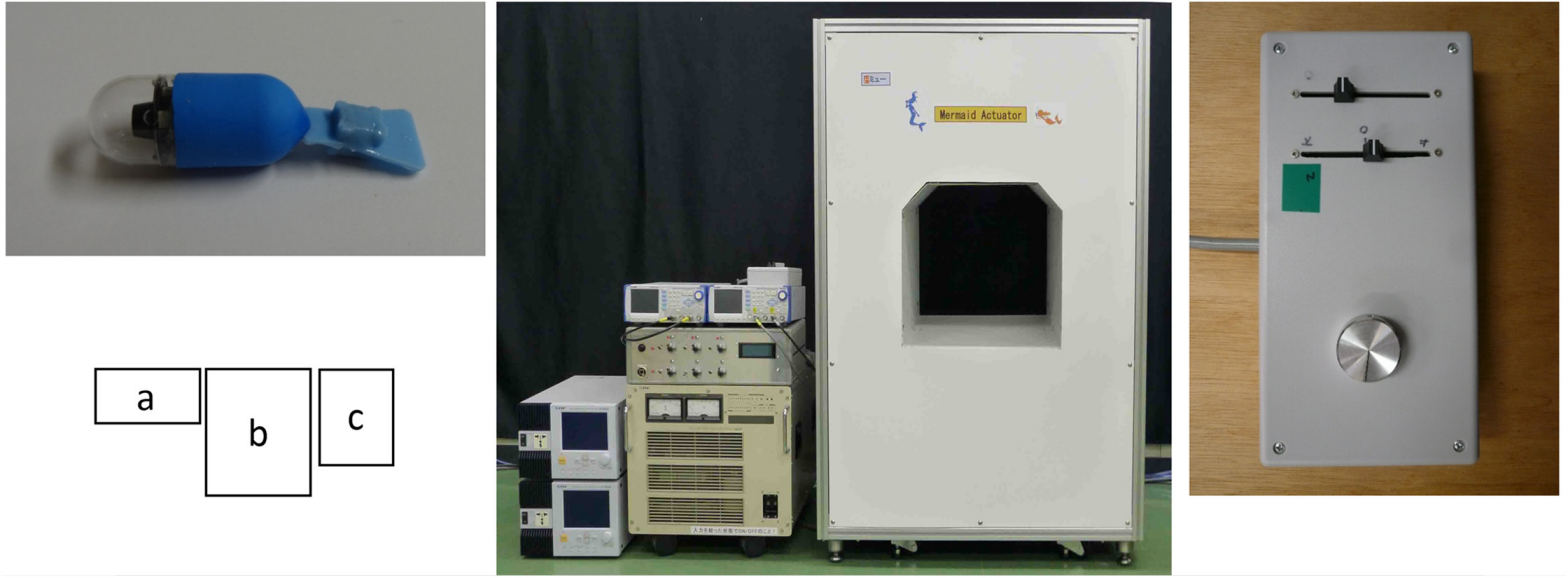
(a) The body of a self-propelling capsule endoscope (SPCE). (b) A patient undergoes SPCE examination using the New MiniMermaid system. (c) The SPCE maneuver is conducted by a controller.

**Fig 2:**
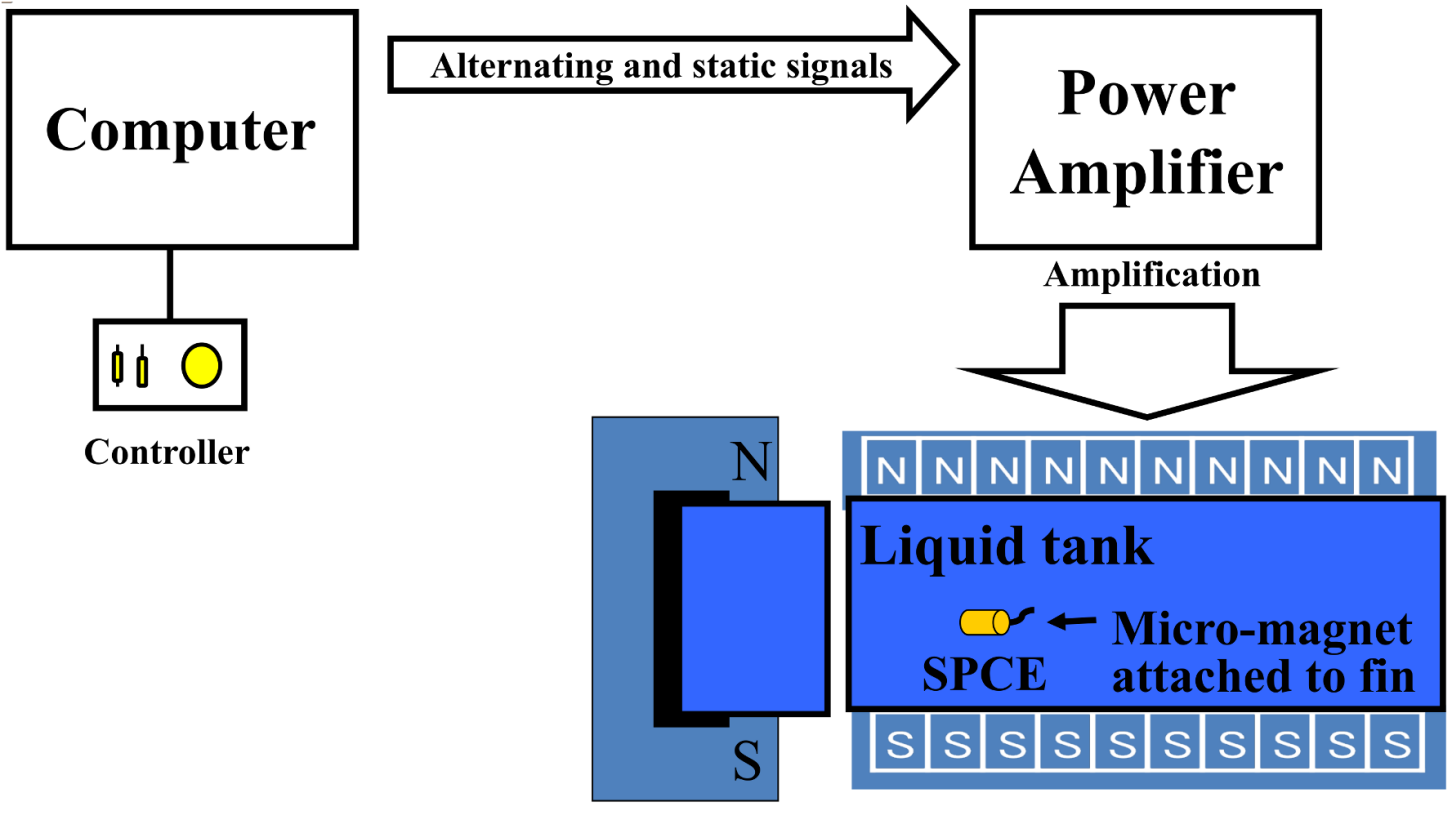
Driving system for the self-propelling capsule endoscope (SPCE), which uses a magnetic field (New MiniMermaid system).

The SPCE was built by connecting a dedicated fin made of silicon resin with a micro-magnet to an existing CE. The length of the fin was 19 mm (Fig 1). When the micro-magnet is placed in an alternating magnetic field, it vibrates. As the vibration transmitted to the fin, it is converted to a propelling force if in water. Therefore, it is necessary to provide water into the stomach for the control of the SPCE. Furthermore, three-dimensional control of the SPCE could be achieved by adjusting the magnetic field. In our experiments, an examiner controlled the SPCE with a dedicated controller while observing via a real-time monitoring system (RAPID Access; Given Imaging Ltd., Israel) (Fig 2) [8].

### SPCE

The three types of CEs used in the present study are commercially available in several countries: PillCamSB2, PillCamESO2, and PillCamCOLON2 (Given Imaging Ltd., Israel) [10–12]. The main specifications of each CE are shown in Table 1. The PillCamSB2, which is used for the small intestine, has a single head and captures images at two frames per second (fps) at a viewing angle of 156°. The PillCamESO2, commonly used for the esophagus, has double heads, which capture images at 18 fps each and provide an extra wide viewing angle of 169°. The PillCamCOLON2 for the colon also has double heads with an adaptive frame rate (AFR) and a viewing angle of 172° [10–12]. The AFR allows the PillCamCOLON2 to capture images at 18 fps in motion mode and two fps in virtually stationary mode [13]. The PillCamCOLON2 floats more easily than the PillCamSB2 and PillCamESO2 owing to its smaller specific gravity [14]. For the double-headed CEs—i.e., the PillCamESO2 and PillCamCOLON2—the camera of one head was used for this experiment, whereas that of the other head was attached to the fin and not used to capture images (Fig 1A). In this study, the three SPCEs attached to the PillCamSB2, PillCamESO2, and PillCamCOLON2 were named as SPCE-SB2, SPCE-ESO2, and SPCE-COLON2, respectively.

**Table 1:** Specifications of the three capsule endoscopes used in the present study.

CE	Type of camera	Frames per second	Viewing angle (degrees)
PillCamSB2	Single camera	2	156
PillCamESO2	Double cameras	18	169
PillCamCOLON2	Double cameras	2 or 18 (AFR)	172

Abbreviations: AFR: adaptive frame rate, CE: capsule endoscope

### Stomach Model

**Fig 3:**
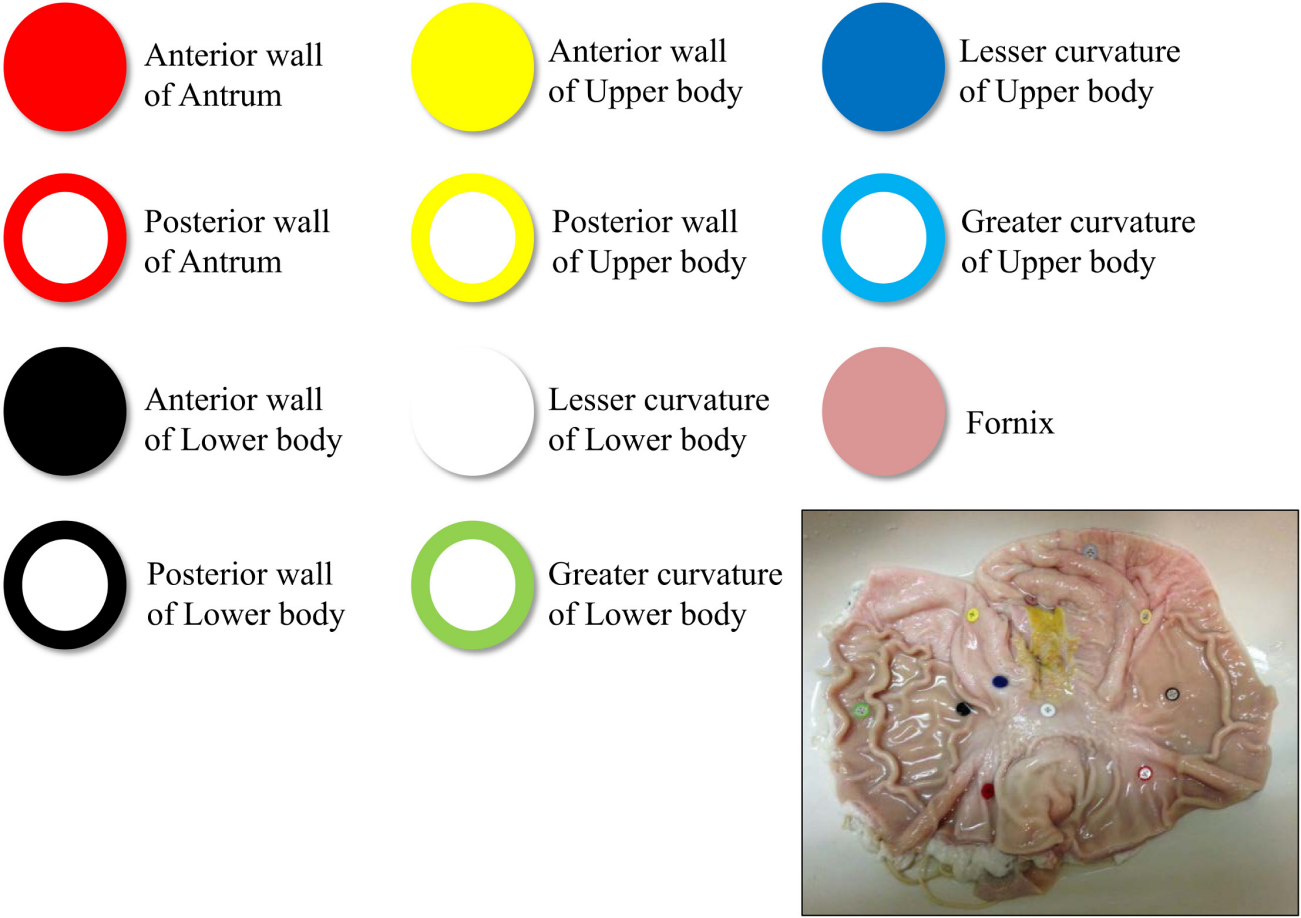
In total, 11 buttons with different colors were sewed to the mucosal side of the porcine stomach model. The image in the lower right panel is the stomach model, which was opened along the greater curvature after the experiment.

**Fig 4:**
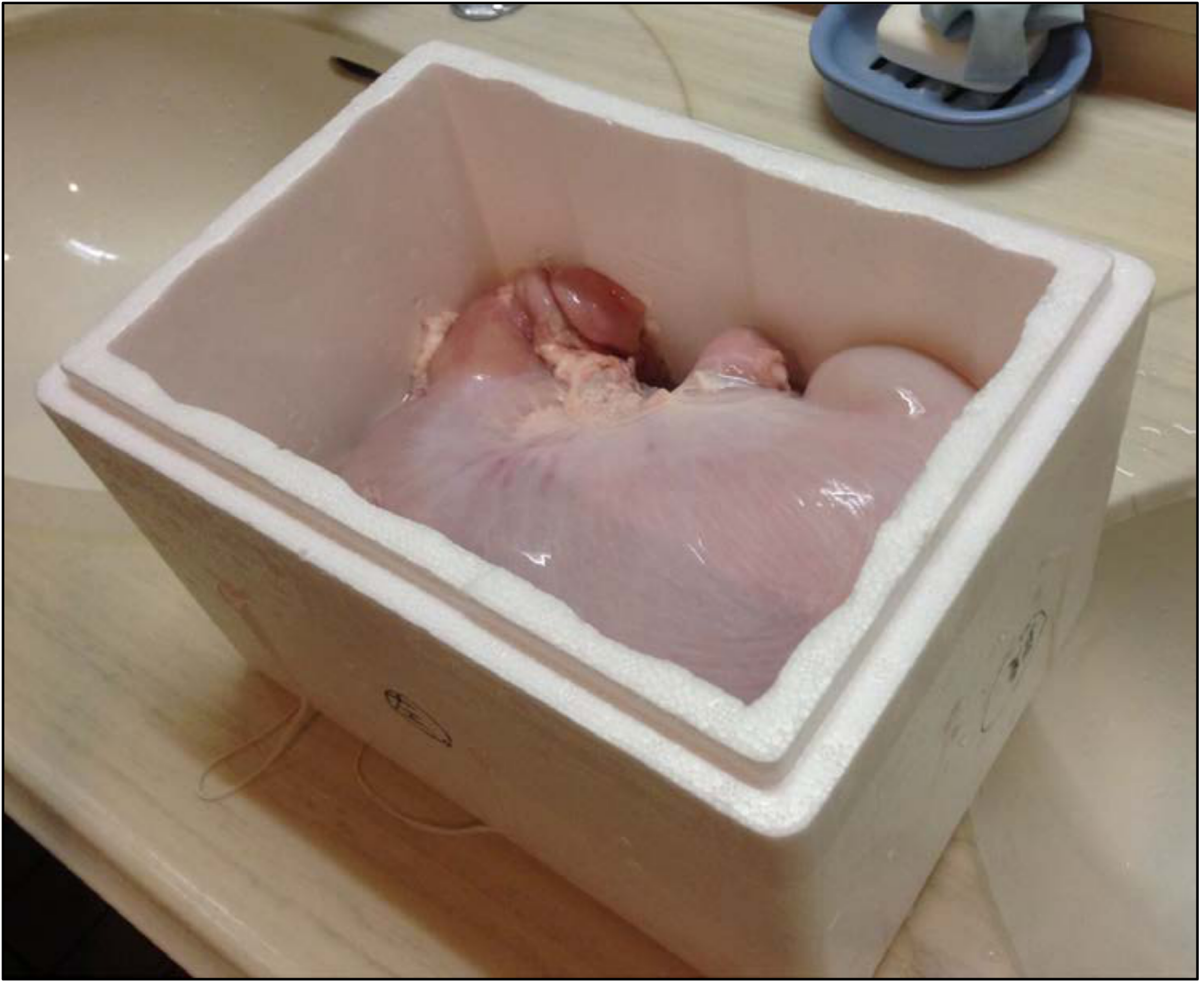
The stomach model was filled with 500 mL of water and fixed in a styrol-foamed box to allow it to rotate manually.

The stomach model was created from an excised porcine stomach. Eleven different colored buttons were sewn on to the mucosal side of the stomach (Fig 3). Each button's color is clearly different from the color of the mucosa of porcine stomach without blood flow. In the human stomach, quantitative evaluation is often difficult because it is not possible to sew buttons as landmarks. The stomach model was fixed in a styrol-foamed box to allow it to rotate manually because a patient is allowed to change postural positions in clinical practice. The stomach model was filled with 500 mL of water before the experiment (Fig 4). The excised porcine stomach was obtained from the Osaka Meat Organ Corporation (Osaka, Japan) two days prior to the procedure. The stomach was prepared as described above the day prior to the procedure.

### Evaluation

Four examiners (KO, TK, MI, and KU) participated in the experiment. The four examiners knew the positions of the colored buttons in the stomach model beforehand. Each examiner controlled the SPCE in the stomach model for 10 min, attempting to detect and closely examine as many buttons as possible. The stomach model was rotated, as per the examiner’s instruction. First, the stomach model was placed supine, and then rotated towards the left lateral decubitus position, the prone position, and finally, the right lateral decubitus position. The reason for the position change was to minimize the amount of water flowing out of the stomach into the duodenum. The examination was conducted ten times using the SPCE-SB2 (KO: 3 times, TK: 3 times, MI: 2 times, KU: 2 times) and SPCE-COLON2 (KO: 3 times, TK: 3 times, MI: 2 times, KU: 2 times). However, for the SPCE-ESO2, the test was performed only nine times because the battery of the PillCamESO2 completely discharged during examination (KO: 3 times, TK: 2 times, MI: 2 times, KU: 2 times).

**Fig 5:**
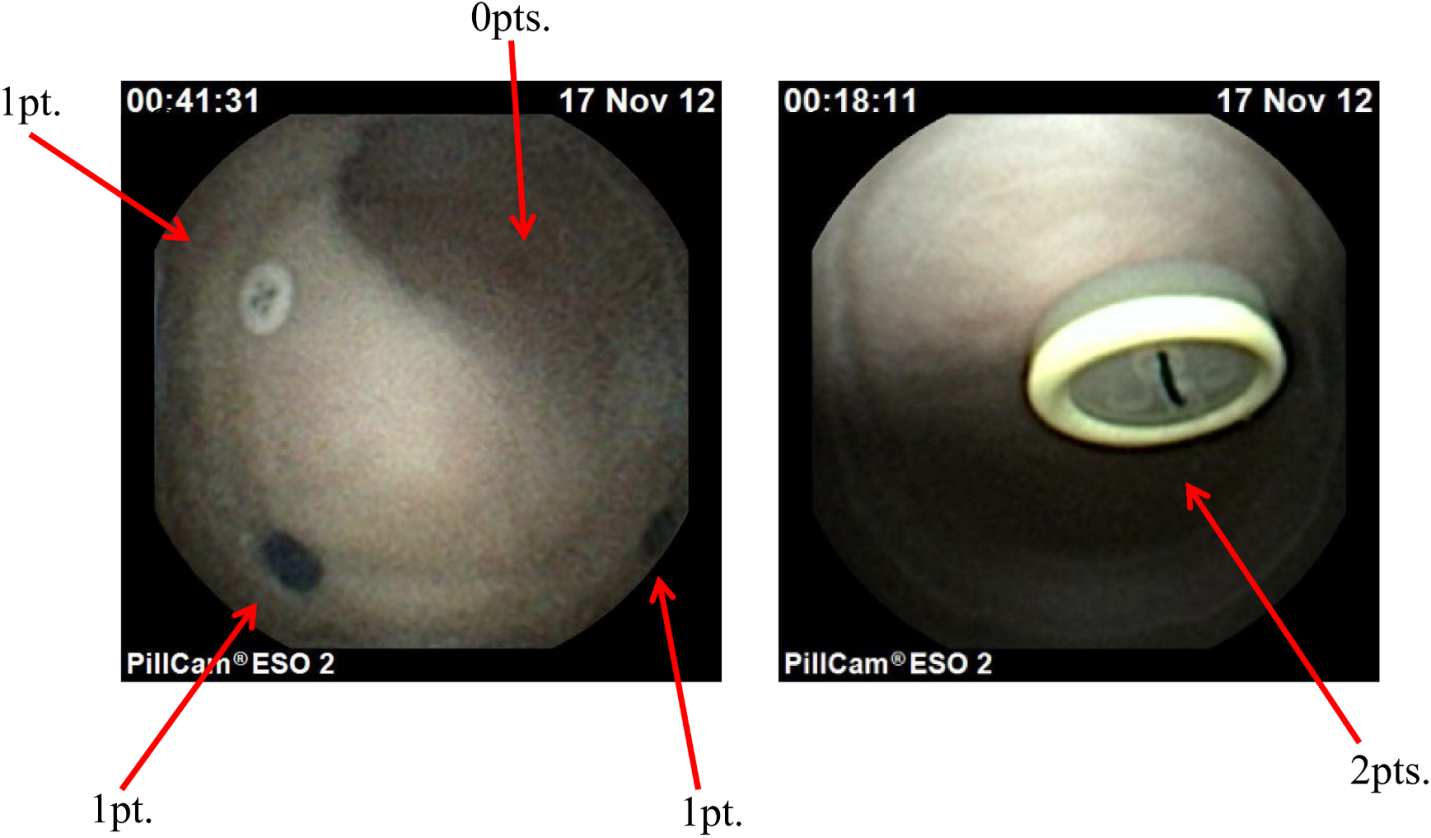
Examples of the images and their associated self-propelling capsule endoscope-performance scores. The score is the sum of the points given to each button as follows: 2 points were given if the buttons could be approached and observed closely; 1 point was given if the buttons came into view but could not be approached closely; and no points were given if the buttons could not be detected.

We devised two parameters to evaluate the detecting capability of the SPCEs. First, the number of detected buttons was used to assess their ability to find lesions. Second, an SPCE-performance score was calculated to evaluate the ability to examine these lesions in detail. The score was defined as the sum of points given to each button as follows: 2 points were given if a button could be approached and observed closely; 1 point was given if a button came into view but could not be approached closely; and no points were given if a button could not be detected (Fig 5). The maximum number of buttons was 11, and that of the SPCE-performance score was 22 points.

The experiment of each SPCE were spaced more than a month, and the examiner underwent an experiment of each SPCE after they practiced several times with SPCE-SB2 in the previous experiment. Thus the improvement of the experimenter SPCE operation by overlaying a number of experiments are not considered.

### Statistical Analysis

The significant differences between the means of data for two different test groups were evaluated by the Mann-Whitney *U*-test. A p-value of <0.05 was considered significant, and all tests were two-sided. Data are expressed as the mean ± standard deviation. All statistical analyses were performed using PASW Statistics 18 for Windows (SPSS Japan, Tokyo).

## Results

**Fig 6:**
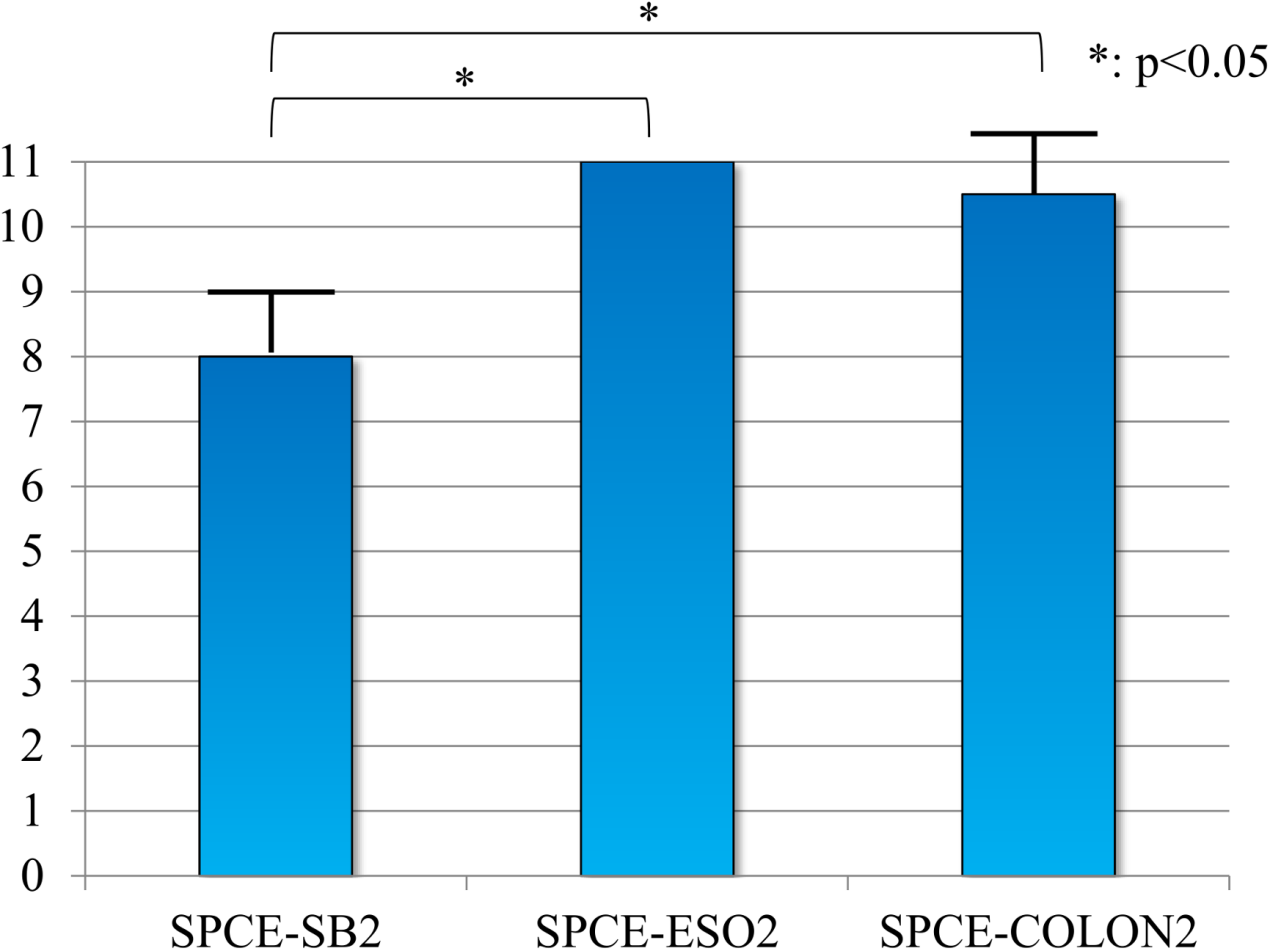
Comparison of the ability to find lesions (the number of buttons detected by the self-propelling capsule endoscope in 10 min). This preliminary test was conducted ten times using the SPCE-SB2 (KO: 3 times, TK: 3 times, MI: 2 times, KU: 2 times) and the SPCE-COLON2 (KO: 3 times, TK: 3 times, MI: 2 times, KU: 2 times); however, for the SPCE-ESO2, the test was performed only nine times because the battery of the PillCam ESO2 completely discharged during the examination (KO: 3 times, TK: 2 times, MI: 2 times, KU: 2 times).

The median number of buttons detected in 10 min was 11 for the SPCE-ESO2 [interquartile range (IQR): 0], 10.5 (0.5) for the SPCE-COLON2, and 8 (1.0) for the SPCE-SB2. The SPCE-ESO2 and SPCE-COLON2 had a significantly better ability to detect lesions than the SPCE-SB2 (p < 0.05). There was no significant difference in the ability to detect lesions between the SPCE-COLON2 and SPCE-ESO2. The examiner was able to detect all the buttons in every examination using the SPCE-ESO2 (Fig 6).

**Fig 7:**
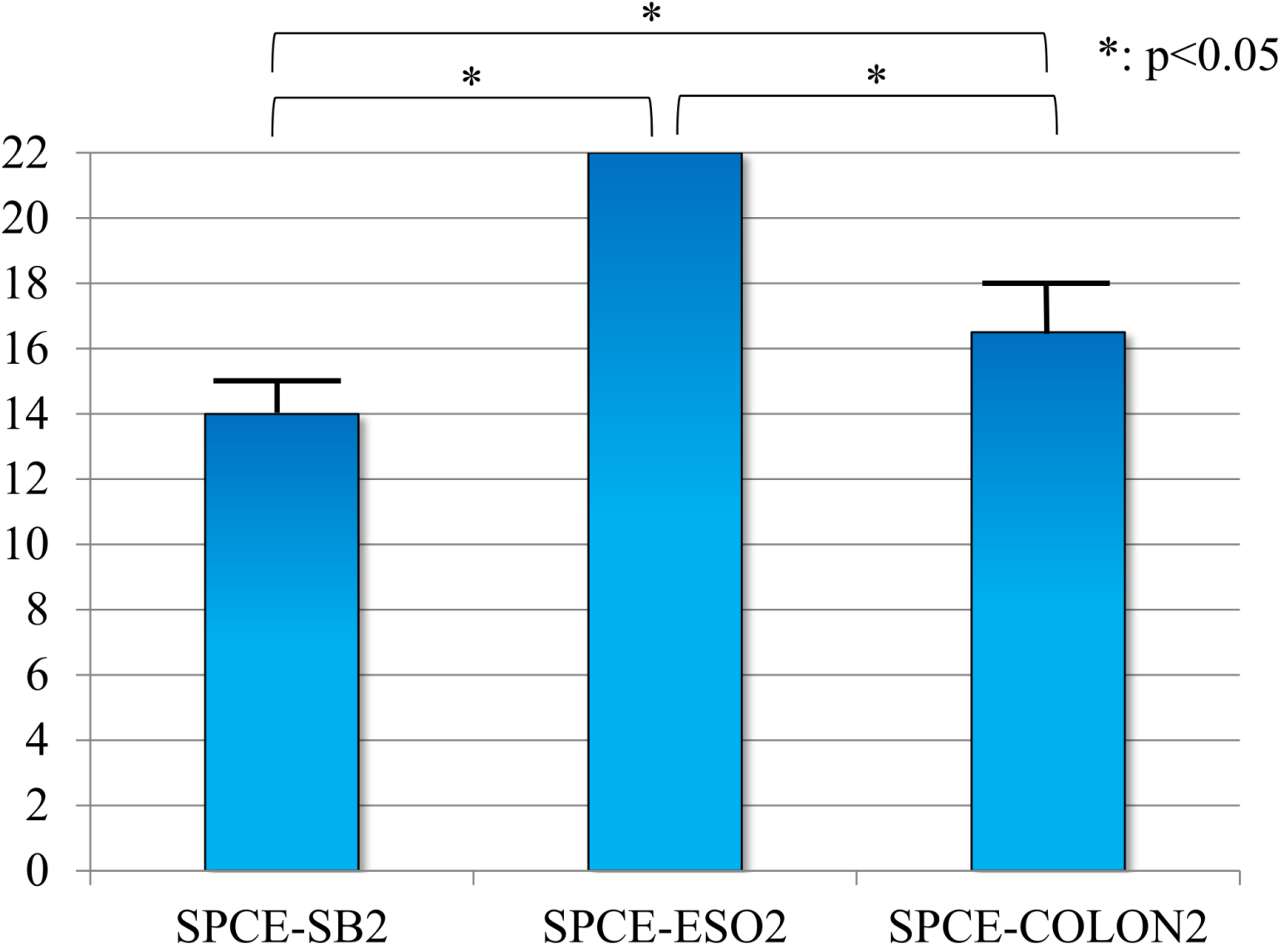
Comparison of the ability to examine the lesions in detail via the self-propelling capsule endoscope-performance scores. This preliminary test was conducted ten times using the SPCE-SB2 (KO: 3 times, TK: 3 times, MI: 2 times, KU: 2 times) and the SPCE-COLON2 (KO: 3 times, TK: 3 times, MI: 2 times, KU: 2 times); however, for the SPCE-ESO2, the test was performed nine times because the battery of the PillCam ESO2 completely discharged during the examination (KO: 3 times, TK: 2 times, MI: 2 times, KU: 2 times).

The median SPCE-performance score was 22 (0) for SPCE-ESO2, 16.5 (1.5) for SPCE-COLON2, and 14 (1.0) for SPCE-SB2. There were significant differences between the three SPCE types (p < 0.05 for all of the comparisons: SPCE-SB2 versus SPCE-ESO2, SPCE-SB2 versus SPCE-COLON2, and SPCE-ESO2 versus SPCE-COLON2) (Fig 7).

In the 10 examinations performed using the SPCE-SB2, detection of the buttons located in the upper body of the greater curvature and the fornix was considerably difficult. The SPCE-SB2 could detect the button at the fornix in only two examinations and the one at the greater curvature of the upper body in three examinations. However, detection of the buttons located in the antrum of the posterior wall (10/10 exams), the lower body of the lesser curvature (9/10), and the upper body of the posterior wall (9/10) was almost always possible.

In eight of the nine examinations performed using the SPCE-ESO2, all the buttons could be observed in detail. The time required for the SPCE-ESO2 to observe all the buttons in detail was 468 ± 74 s (7.8 ± 1.2 min).

The SPCE-performance score of the SPCE-SB2 was low at 14 (Table 2). Of the three SPCE types, the SPCE-ESO2 was the most effective in detecting lesions and evaluating them closely (S2–S4 Videos).

**Table 2:** Detecting capability of the three self-propelling capsule endoscope types.

SPCE	-SB2	-ESO2	-COLON2
Number of the examination times	10	9	10
The ability to find lesions (the total number of detected buttons, maximum number: 11)	7.6 ± 1.1	11 ± 0	10.1 ± 1.0
The ability to examine lesions in detail (the SPCE-performance score, maximum value: 22)	13.9 ± 1.6	21.9 ± 0.3	16.6 ± 2.1

Abbreviation: SPCE: self-propelling capsule endoscope

## Discussion

The present study identified the CE specifications that affected the SPCE functions. The SPCE-COLON2 and SPCE-ESO2 had a larger viewing angle than the SPCE-SB2, and were able to detect significantly more buttons than the latter one within 10 minutes, which was the primary reason for the enhanced ability to find lesions. In addition, SPCE-COLON2 and SPCE-ESO2, which could take several images in a second, were able to approach closer to the buttons than the SPCE-SB2.

By using a porcine stomach model, we demonstrated that the most suitable CE for SPCE was the PillCamESO2. Since the video obtained from the SPCE-ESO2 appeared in a continuous manner, maneuvering the device and approaching the buttons inside the model were easy via wireless control from outside of the stomach model. However, we did not expect that the SPCE-COLON2 would be inferior to the SPCE-ESO2. Because the PillCamCOLON2 is designed for the observation of the colon, its specific gravity is lower, and the device is more buoyant than the PillCamESO2 [14]. Therefore, the PillCamCOLON2 tends to face upward rather than downward in the water. Moreover, the SPCE-COLON2 does not send all captured images to the real-time monitoring system, whereas the SPCE-ESO2 could send all the images captured at 18 fps. Furthermore, the capturing speed of the SPCE-COLON2 is not constant owing to the AFR. These factors were reflected in the different SPCE-performance scores obtained in our experiments. The higher capturing speed enabled fine adjustments while controlling the SPCE. In other words, the examiner was able to move the SPCE more accurately towards the target, approach it, and stop at an appropriate distance.

Our results suggested that the SPCE-ESO2 could be used to screen and closely examine the human stomach in less than 10 min, which is within the usual duration of a gastric endoscopy procedure and allows for sufficient examination before the PillCamESO2 runs out of battery. However, the SPCE-SB2, with an inferior performance compared to the SPCE-ESO2, might miss those lesions located in the upper body of the greater curvature and the fornix. In addition, we found that lesions in the antrum of the posterior wall, the lower body of the lesser curvature, and the upper body of the posterior wall were easy to detect by using the SPCE. The SPCE could easily approach close to the posterior wall when the magnetic field was weakened. Observation of the fornix seemed to be difficult because the SPCE required to be maneuvered over large folds around the fornix, where air tends to accumulate. It is important to identify these possible blind sites before clinical application. Nonetheless, real stomach lesions cannot be compared to the buttons in the stomach model used in this study. Therefore, we need to assess the observation sequence more efficiently by performing further experiments using the stomach model. Rey et al. reported the results of clinical trial using Magnetically Guided Capsule Endoscopy (MGCE) which was a capsule endoscope operatable from outside of the body developed by themselves but this was not a comparison examination of capability of MGCE [15, 16]. From our results, for clinical application, it is considered that a controllable CE with functions such as SPCE-ESO2 is suitable.

Our study has several limitations. The stomach model in this study is not the same as a live human stomach with peristaltic motion and the secretion of gastric mucus. Also, the pig stomach model mucosa lacks blood flow, and is whiter than the gastric mucosa of a live human. In addition, the four examiners knew the position of the colored buttons in the stomach model and this may have made the detection of the colored buttons in the stomach model easier. However, we objectively compared three types of SPCE and demonstrated for the first time that SPCE could observe every area of the stomach. Because sewing colored buttons to an in vivo stomach is not practical, we chose to use the excised porcine stomach. We demonstrated that the SPCE-ESO2 could be used to examine the entire internal surface of the excised porcine stomach. Future studies in the live human stomach are necessary.

## Conclusions

In the present study, we demonstrated that the ability to find lesions and examine them in detail depended on the CE viewing angle and the frame rate of the images, respectively. These results might allow us to solve other problems of the SPCE, such as controlling the CE from outside the body at will, finding a lesion, and examining it in detail. The SPCE-ESO2 might be the most feasible in clinical practice. Future experiments using the SPCE-ESO2 in a human body are warranted.

Additionally, our results are considered to be universal for the development of a CE that is controllable from outside of the body and allows for real-time observation, including SPCE.

## Supporting Information

S1 Video The self-propelling capsule endoscope is controllable in three-dimensional directions if in water. (ZIP)

S2 Video The view of the SPCE-SB2. (ZIP)

S3 Video The view of the SPCE-ESO2. (ZIP)

S4 Video The view of the SPCE-COLON2. (ZIP)

## References

[1] Iddan G, Meron G, Glukhovsky A, Swain P. Wireless capsule endoscopy. Nature. 2000; 405: 417. 10.1038/35013140 10839527

[2] Eliakim R, Sharma VK, Yassin K, Adler SN, Jacob H, Cave DR, et al A prospective study of the diagnostic accuracy of PillCam ESO esophageal capsule endoscopy versus conventional upper endoscopy in patients with chronic gastroesophageal reflux diseases. J Clin Gastroenterol. 2005; 39: 572–8. 16000923 10.1097/01.mcg.0000170764.29202.24

[3] Eliakim R, Fireman Z, Gralnek IM, Yassin K, Waterman M, Kopelman Y, et al Evaluation of the PillCam Colon capsule in the detection of colonic pathology: results of the first multicenter, prospective, comparative study. Endoscopy. 2006; 38: 963–70. 17058158 10.1055/s-2006-944832

[4] Kakugawa Y, Saito Y, Saito S, Watanabe K, Ohmiya N, Murano M, et al New reduced volume preparation regimen in colon capsule endoscopy. World J Gastroenterol. 2012; 18: 2092–8. 10.3748/wjg.v18.i17.2092 22563197 PMC3342608

[5] Kurokawa S, Katsuki S, Fujita T, Saitoh Y, Ohta H, Nishikawa K, et al A randomized, double-blinded, placebo-controlled, multicenter trial, healing effect of rebamipide in patients with low-dose aspirin and/or non-steroidal anti-inflammatory drug induced small bowel injury. J Gastroenterol. 2014; 49: 239–44. 10.1007/s00535-013-0805-2 23595613

[6] Matsushita Y, Narahara Y, Fujimori S, Kanazawa H, Itokawa N, Fukuda T, et al Effects of transjugular intrahepatic portosystemic shunt on changes in the small bowel mucosa of cirrhotic patients with portal hypertension. J Gastroenterol. 2013; 48: 633–9. 10.1007/s00535-012-0660-6 22968470

[7] Fukumoto A, Tanaka S, Shishido T, Takemura Y, Oka S, Chayama K. Comparison of detectability of small-bowel lesions between capsule endoscopy and double-balloon endoscopy for patients with suspected small-bowel disease. Gastrointest Endosc. 2009; 69: 857–65. 10.1016/j.gie.2008.06.007 19136103

[8] Morita E, Ohtsuka N, Shindo Y, Nouda S, Kuramoto T, Inoue T, et al In vivo trial of a driving system for a self-propelling capsule endoscope using a magnetic field (with video). Gastrointest Endosc. 2010; 72: 836–40. 10.1016/j.gie.2010.06.016 20883863

[9] Ohtsuka N, Umegaki E, Shindo Y, Uesugi K, Nishihara H, Nouda S, et al Observation of whole digestive tract of a human by a single passage of a self-propelling capsule endoscope. Gastrointest Endosc. 2012; 75: AB126.

[10] Selby WS, Prakoso E. The inability to visualize the ampulla of Vater is an inherent limitation of capsule endoscopy. Eur J Gastroenterol Hepatol. 2011; 23: 101–3. 10.1097/MEG.0b013e3283410210 21030868

[11] Metzger YC, Adler SN, Shitrit AB, Koslowsky B, Bjarnason I. Comparison of a new PillCam^TM^ SB2 video capsule versus the standard PillCam^TM^ SB for detection of small bowel disease. Rep Med Imaging. 2009; 2: 7–11.

[12] Spada C, De Vincentis F, Cesaro P, Hassan C, Riccioni ME, Minelli Grazioli L, et al Accuracy and safety of second-generation PillCam COLON capsule for colorectal polyp detection. Therap Adv Gastroenterol. 2012; 5: 173–8. 10.1177/1756283X12438054 22570677 PMC3342572

[13] Kakugawa Y, Matsumoto M, Saito Y. Clinical practice using colon capsule endoscopy. Nihon Rinsho. 2014; 72: 168–74. 24597367

[14] Valdastri P, Simi M, Webster RJ 3rd. Advanced technologies for gastrointestinal endoscopy. Annu Rev Biomed Eng. 2012; 14: 397–429. 10.1146/annurev-bioeng-071811-150006 22655598

[15] Rey JF, Ogata H, Hosoe N, Ohtsuka K, Ogata N, Ikeda K, et al Blinded nonrandomized comparative study of gastric examination with a magnetically guided capsule endoscope and standard videoendoscope. Gastrointest Endosc. 2012; 75: 373–81. 10.1016/j.gie.2011.09.030 22154417

[16] Rey JF, Ogata H, Hosoe N, Ohtsuka K, Ogata N, Ikeda K, et al Feasibility of stomach exploration with a guided capsule endoscope. Endoscopy. 2010; 42: 541–5. 10.1055/s-0030-1255521 20593331

